# A Case Study of Behaviour and Performance of Confined or Pastured Cows During the Dry Period

**DOI:** 10.3390/ani6070041

**Published:** 2016-07-13

**Authors:** Randi A. Black, Peter D. Krawczel

**Affiliations:** Department of Animal Science, University of Tennessee, Knoxville, TN 37996, USA; rblack12@vols.utk.edu

**Keywords:** dairy cattle, dry cow management, behaviour

## Abstract

**Simple Summary:**

Pasture and freestall systems offer benefits and consequences during lactation but have not been investigated during the dry period. The effect of pasture or confined systems during the dry period on behaviour and milk quality was investigated. Freestall housing resulted in more resting behaviour and less locomotor activity during the dry period compared to pastured cows. At calving, freestall housed cows performed fewer lying bouts and less locomotor activity compared to pastured cows. Pasture resulted in less aggression around feeding but high respiration rates during peak heat times. Pasture during the dry period altered lying behavior, reduced feed bunk aggression and increased heat stress behaviors.

**Abstract:**

The objectives of this study were to determine the effect of the dry cow management system (pasture or confined) on: (1) lying behaviour and activity; (2) feeding and heat stress behaviours; (3) intramammary infections, postpartum. Non-lactating Holstein cows were assigned to either deep-bedded, sand freestalls (*n* = 14) or pasture (*n* = 14) using rolling enrollment. At dry-off, cows were equipped with an accelerometer to determine daily lying time (h/d), lying bouts (bouts/d), steps (steps/d) and divided into periods: far-off (60 to 15 d prepartum), close-up (14 to 1 d prepartum), calving (calving date) and postpartum (1 to 14 d postpartum). Respiration rates were recorded once weekly from dry off to calving from 1300 to 1500 h. Feeding displacements were defined as one cow successfully displacing another from the feed bunk and were recorded once per week during the 2 h period, immediately after feeding at 800 h. Pastured cows were fed a commercial dry cow pellet during far-off and total mixed ration during close-up, with free access to hay and grazing. Freestall housed cows were fed a total mixed ration at far-off and close-up. Cows housed in freestalls were moved to a maternity pen with a mattress at commencement of labour. Pastured cows calved in pasture. After calving, all cows were commingled in a pen identical to the freestall housing treatment. Cows housed in freestalls laid down for longer during far-off and close-up periods, had fewer lying bouts during the calving period and took fewer steps throughout the study period when compared to pastured cows. Freestall housed cows experienced more displacements after feeding than did pastured cows. Respiration rates increased with an increasing temperature humidity index, more in pastured cows than in freestall housed cows. Pastured cows altered their lying behaviour and activity, suggesting a shift in time budget priorities between pastured and confined dry cows. Pastured cows also experienced less aggression around feeding but may be more susceptible to heat stress.

## 1. Introduction

Sixty percent of dairy producers in the United States house dry cows separately from lactating cows [1] with the majority of operations housing dry cows in tie-stalls or stanchions (18.2%), freestalls (29.3%), pasture (11.3%), or dry-lots (29.2%) [2]. Though a large number of dairy operations use tie-stalls and stanchions for dry cows, they are more common in smaller dairies (fewer than 100 cows; 57.4% of operations) than large dairies (500 cows or more; 0% operations) and fewer tie-stall facilities are being constructed compared to freestall [2]. Freestalls and pasture present a modern and abundant dry cow housing option that allow for more freedom of movement [3]; however no data exists directly comparing the differences of these two systems during the dry period.

Although no research has been conducted comparing confinement and pasture during the dry period, research has investigated their differences during lactation. Cows in freestall housing fed a corn silage-based total-mixed ration had greater DMI and milk production [4,5,6], lost less body weight postpartum [4,7] and maintained higher levels of plasma glucose postpartum [4], compared to cows managed on pasture eating a grass-based diet. However, pasture systems allow for improved gait and hoof health compared to freestalls due to time on concrete [6,8]. Further, when given a choice between pasture and freestalls, cows prefer to lie on pasture [9], as freestalls can cause abnormal lying and standing up behaviours due to stall partitions [10]. This preference changes during times of heat stress and increasing temperature-humidity index (THI) where cows prefer to be indoors [9], suggesting a preference for freestalls during warmer weather. However, cows displayed a preference to lie outdoors during the night hours [9], which may not compromise DMI or milk production [11].

Pastured cows also had improved udder health over confined cows [7,12,13]. The dry period represents a time when a significant number of intramammary enterobacterial infections were acquired and were more likely to develop into mastitis postpartum [14]. The importance of maintaining udder health during the dry period to prevent intermammary infections has been well established [15,16,17] and housing may play a significant role in intramammary infection acquisition. A reduction in bacterial exposure during the dry period may be effective in minimizing postpartum intramammary infection and improving performance during lactation.

These studies indicate that both pasture and freestall management systems provide benefits and consequences during the lactating period that may be similar during the dry period, particularly differences in lying behaviours due to preferences to lie on pasture or freestalls due to environmental factors. However, understanding what benefits and consequences exist during the dry period between the two systems have not been investigated. Therefore, the objectives of this study was to determine the effects of the dry cow management system (pasture or confined) on (1) lying behaviour and activity; (2) feeding and heat stress behaviours; (3) intramammary infections postpartum.

## 2. Materials and Methods

### 2.1. Animals and Management

All animal procedures were approved by the University of Tennessee IACUC committee (IACUC Protocol #2198-0713). Twenty-eight non-lactating Holstein dairy cows were assigned to either freestall housing (primiparous = 10; multiparous = 5) or pasture (primiparous = 8; multiparous = 7) at dry-off using rolling enrollment from August 2013 to January 2014. Cows entered treatment groups throughout the study period depending on their dry-off date, which was 60 d before their projected calving date and ranged from August to November 2013. Cows were balanced by previous milk production (36.9 ± 6.0 kg/d), parity (1.5 ± 0.6) and projected calving date. A 60-d dry period (59.2 ± 4.6 d) was used and cows were divided into a far-off group (dry off to 2 weeks before projected calving date) and a close-up group (two weeks before projected calving to calving) in both treatments.

**Figure 1:**
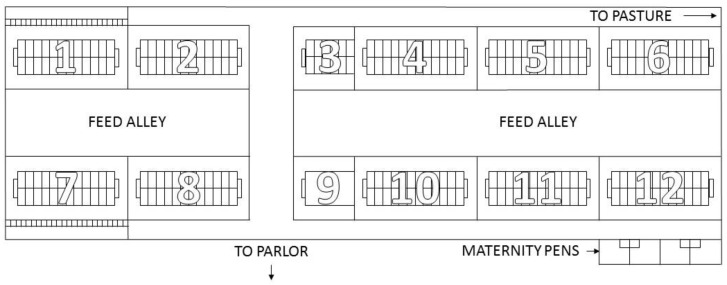
Diagram of University of Tennessee’s Little River Animal and Environmental Unit freestall dairy barn. Study cows were managed in pen 12 due to the close proximity to the maternity stalls.

Freestall housing consisted of a naturally ventilated, 4-row freestall barn (Figure 1) at the University of Tennessee’s Little River Animal and Environmental Unit (Walland, TN, USA). Deep-bedded sand freestalls were 1.2 m wide and 2.4 m long with a neck rail 1.2 m above sand bedding, placed 1.7 m from the curb and a 0.6 m high PVC tube brisket board placed 1.7 m from the curb. New sand bedding was added to stalls once per week and stalls were raked clean twice daily while the lactating herd was milked (0730 to 0930 h and 1730 to 1930 h). Study cows were kept in a pen measuring 19.4 m long and 12.1 m wide containing 24 freestalls and 26 headlocks, with headlocks measuring 0.6 m wide. The pen contained two waterers (Rotary Flush Waterer, Sturdy Built Manufacturing, Denver, PA, USA), 3.3 m long and 0.3 m wide, with one on each end of the pen, and grooved concrete alleyways. Stocking density was calculated as; the number of cows in the pen divided by the number of headlocks with feed available, assuming one cow per headlock as 100% [3]. When there were cows in both the far-off and close-up periods, the pen was divided in half with 12 freestalls, 13 headlocks and one waterer in each pen. Freestall housing was equipped with fans every 7.2 m above the freestalls and headlocks. Fans turned on automatically when the temperature rose above 23 °C. During the far-off period, the group size ranged from 2 to 9 with a mean of 5.1 ± 2.0 cows, with feed bunk stocking density ranging from 22.2% to 69.2% with a mean of 41.1% ± 13.8%. During the close-up period, the group size ranged from 2 to 3 with a mean of 2.4 ± 0.5 cows while feed bunk stocking density ranged from 15.8% to 60.0% with a mean of 31.8% ± 15.4%. As freestalls were not the typical dry cow management strategy of the farm, only study cows were added to the pens weekly at dry-off, if applicable. 

**Figure 2:**
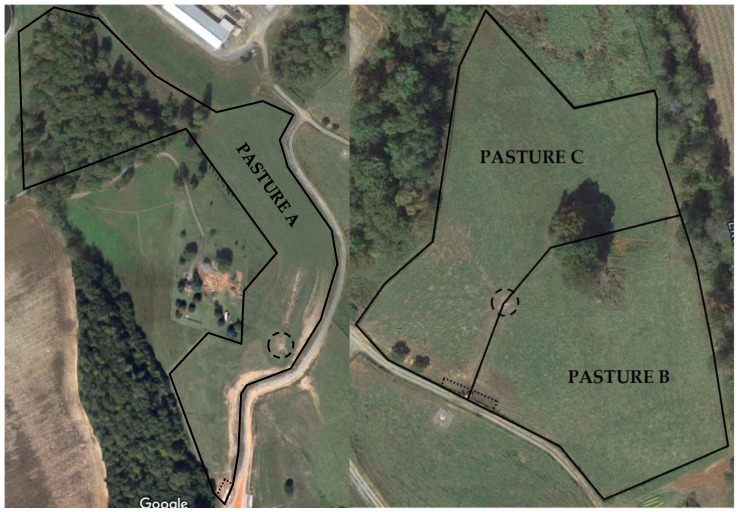
Aerial view of paddocks at University of Tennessee’s Little River Research and Environmental Unit. Cows were managed in Pasture A from August to September 2013 and Pastures B and C from September 2013 to January 2014. Water locations are denoted by a dashed circle and feedbunk locations are denoted by dotted lines. Aerial images obtained through Google Maps (Google, Moutain View, CA, USA).

Between August and September 2013, pastured cows were moved into a 13 acre pasture (Pasture A; Figure 2), comprising of two combined pastures; the first 427.5 m at the longest points and 206.1 m at the widest points; and the second measuring 162.0 m at the longest points and 139.9 m at the widest points. The pasture contained one waterer (Thrifty-King CT4-2000, Ritchie Industries, Inc., Conrad, IA, USA), with four 30.5 cm diameter ball openings and 6 concrete feed bunks measuring 2.5 m long, 0.8 m deep and 0.5 m wide each, totaling 15 m of vertical feed bunk space. Cows had continuous access to hay (Table 1) available with open access on the ground. Stocking density was calculated as; the number of cows in the pen divided by the amount of bunk space with feed available, assuming one cow per 0.6 m bunk space as 100% [3]. Study cows, only far-off cows during this time, were comingled with non-study far-off dry cows and heifers 4 week from calving, with a group size ranging from 6 to 15 and a mean of 9.2 ± 4.2 cows with a feed bunk stocking density ranging from 36.6% to 85.3%, with a mean of 57.6% ± 20.0%. New cows and heifers were added once a week, if applicable. Cows were managed on a different pasture from September 2013 to January 2014 due to the need for adjacent close-up and far-off pens for behavioural observations. Pens were; a 2.8 acre far-off pen (Pasture B; Figure 2), measuring 131.2 m at the longest points and 131.0 m at the widest points; and a 4.3 acre close-up pen (Pasture C; Figure 2), measuring 189.7 m at the longest points and 140.0 m at the widest points. Both pens shared a common waterer (Thrifty-King CT4-2000, Ritchie Industries, Inc., Conrad, IA, USA), with four 30.5 cm diameter ball openings, with access to two ball openings from each side. Cows in both groups were fed at adjacent feed bunks with each group having access to 2.5 concrete bunks measuring 2.5 m long, 0.8 m deep, and 0.5 m wide each, totaling 6.25 m of vertical bunk space per pen. Cows had continuous access to hay (Table 1) from an elevated hay rack that was located next to the feed bunk and accessible from both pens. During the far-off period, the group size ranged from 5 to 15 with a mean of 7.9 ± 2.8 cows, while feed bunk stocking density ranged from 61.0% to 182.9.0%, with a mean of 98.0% ± 34.0%. During the close-up period, the group size ranged from 2 to 6 with a mean of 4.2 ± 1.5 cows, while feed bunk stocking density ranged from 24.4% to 91.4%, with a mean of 57.0% ± 22.7%. Far-off dry cows not in the study were added to the far-off pen and close-up cows and heifers 4 weeks from calving were added to the close-up pen weekly, when applicable. All pastures had access to natural shade using trees along the perimeter of the pastures.

When cows exhibited signs of calving in the freestall housing treatment (i.e., restlessness, holding of tail, water breaking, swollen vulva), cows were moved into a 4.2 m × 4.1 m maternity pen at night containing a rubber filled mattress covering the entire pen floor (ProMat, Inc., Woodstock, ON, Canada) with no bedding. Cows had access one side of a 30.5 cm diameter ball waterer (Thrifty-King CT2-2000, Ritchie Industries, Inc., Conrad, IA, USA) in the maternity pen and were moved back into the freestall group during the day to access feed. Pastured cows were left to calve on pasture and were moved to the freestall barn when farm staff after the calf was born. After calving, all study cows were moved to a fresh cow group until antibiotic residues were no longer present in their milk and were subsequently moved into the multiparous cow group.

### 2.2. Feeding Management

Samples of pre-mix hay and corn silage were collected from 12 random locations throughout the bale or bunker and thoroughly mixed to create a composite sample of each. Samples were then submitted to a forage testing laboratory for nutrient analysis (Dairy One, Ithaca, NY, USA). Grain A was a commercial feed pelleted by a local feed mill (Tennessee Farmer’s Cooperative, Lavergne, Tennessee), while Grain B was a pellet formulated for the farm’s lactating cows and pelleted by the same local feed mill. Nutrient analyses are presented in Table 1. Freestall housed cows were fed 7.9 kg pre-mix hay, 2.3 kg corn silage, and 2.7 kg grain A combined in a TMR during the far-off period and 5.7 kg pre-mix hay, 9.1 kg corn silage, 1.8 kg grain A and 1.8 kg grain B combined in a TMR during the close-up period. Pastured cows were fed 3.2 kg grain A during the far-off period and 5.7 kg pre-mix hay, 9.1 kg corn silage, 1.8 kg grain A and 1.8 kg grain B combined in a TMR during the close-up period. Both treatments were fed once daily at 800 h.

Pastured cows also had continuous access to grazing throughout the dry period. Pastures were seeded with orchardgrass and KY-31 fescue and grass levels were managed by the farm manager for a height of 0.3 to 0.5 m. Grass samples were collected weekly by placing a 30.5 cm^2^ PVC square at 10 random areas within each pasture and clipping all grass within the square down to 2.5 to 4 cm from soil to cut. All grass samples were combined to create a composite pasture and day sample. Grab samples of hay available to pastured cows were collected from 10 random sites throughout the round bale and thoroughly mixed to create a composite day sample. Grass and hay samples were dried at 55 °C for 48 h and then ground to 1 mm. Samples were analyzed for nutrient composition using near-infrared spectroscopy technology (FOSS 5000, FOSS NIRSystems, Inc., Eden Prairie, MN, USA) as previously described [18].

### 2.3. Behavioural Assessment

Cows were fitted with an IceTag activity monitor (IceRobotics, Edinburgh, UK) at the time of dry off, which was removed 14 d postpartum. One cow was excluded from analysis due to the IceTag malfunctioning; only the first day of data was recorded. Steps, lying bouts and lying time were recorded and averaged by day, with all lying bouts less than 2 min removed [19]. Data was further averaged into four periods: far-off, close-up, calving (calving date; midnight to midnight on date of calving as no video available to determine actual calving time) and postpartum (1 to 14 days postpartum). Periods were created to determine behavioral changes when cows were in different environments (far-off vs. close-up pens, dry cow environment vs. postpartum freestall housing, etc.) and the monitoring of these changes between treatments.

Feed bunk displacements were continuously monitored during the 2-h period after feed delivery at 800 h once weekly from dry-off to calving. Once weekly measures were performed due to the length of the study (5 months) and because previous research has successfully monitored social behavior in cows with two site visits and 8 total h of observation per farm [19]. The current research provides between 12 and 18 h of feed bunk displacement behavior per cow (depending on dry period length) during the period when the majority of feed bunk displacements occur [20]. Displacements were observed when an aggressive behaviour (head butt or push) from one cow (actor) resulted in the complete withdrawal of another cow (reactor) from the headlock (freestall) or feed rail (pasture) [21]. Every successful interaction *(*i.e., reactor cow actually displaced and not just an aggressive interaction) with at least one study cow was recorded. A single observer consistently monitored displacements within the freestall barn while a different observer consistently monitored displacements on pasture, both watching from a distance that did not disturb feeding. The number of displacements were recorded and summarized as the total number within each 2-h period.

Respiration rates were recorded once weekly from dry off to calving at 10-min intervals from 1300 to 1500 h to capture periods where temperatures may have led to heat stress during the warmer months [22,23]. Observations were performed once a week due to the length of the study and because previous research has successfully quantified respiration rates with 30 to 40 observations per cow over 5 to 20 consecutive days [24,25]. In the current study, each cow had between 720 and 1080 respiration rate observations (depending on dry period length) collected during the study period, during peak heat times. The number of breaths, as noted by rise and fall of the flank, were counted over 1 min to determine breaths per min. Respiration rates were measured using a consistent cow order to ensure that each cow had a respiration rate recorded at even 10 min intervals. A single observer consistently monitored freestall housed cows while another single observer consistently monitored pastured cows throughout the entire study period. The observer monitoring freestall housed cows remained at the feedbunk and did not enter the pen unless the rise and fall of the flank was not visible. Then, the observer maintained a distance from the cow so as to not induce stress or falsely increase the cow’s respiration rate. Similarly, the observer monitoring pastured cows maintained a distance that did not impose additional stress or invoke a flight response in cows while monitoring respiration rates. Respiration rates were averaged by each 2 h period for each cow. Outdoor ambient temperature was measured using a weather station (UT10, Campbell Scientific Inc., Logan, UT, USA) located on a small rise at the north end of the freestall barn 73 m from the end of the building. Indoor ambient temperature was measured using a data logger (HOBO U10 Temperature Relative Humidity Data Logger, Onset Computer Corporation, Bourne, MA, USA) located in the middle of the freestalls pen, 1.8 m above the ground to keep from cow reach. Data were summarized by the same 2 h period for each day of respiration rate observation, for outdoor and indoor environments. Temperature humidity index was then calculated for both environments on each observation day and reported in Table 2.

### 2.4. Intramammary Infections

Prior to milking, a composite aseptic milk sample was collected from each quarter into one 15 mL collection vial on d 0, 1 and 2 postpartum [26]. Samples were frozen in a 0 °C freezer until further analysis. Samples were prepared by creating serial dilutions using 0.5 mL of sample to 4.5 mL of phosphate buffer saline in a 1:10 dilution up at a 1:10^2^ dilution. The mixture was vortexed until well mixed. For isolation of gram-negative species, one milliliter of the sample and each dilution was added to two separate MacConkey agars (Remel, Lenexa, KS, USA), prepared according to the manufacturer’s directions. For isolation of *Streptococcus agalactiae*, one milliliter of the sample and each dilution was added to two separate Modified Edwards agars (Remel, Lenexa, KS, USA), prepared according to the manufacturer’s directions. For isolation of coagulase positive *staphylococci*, one milliliter of sample and each dilution were added to two separate Baird-Parker agars (Remel, Lenexa, KS, USA), prepared according to the manufacturer’s directions. A smooth sterilized spreader was used to spread the material across each plate surface. Plates were incubated at 35 °C and 5% CO_2_ for 48 h and colony-forming units were counted manually.

### 2.5. Descriptive Measurements

Descriptive measurements, including cow-based assessments and physiological measures, were collected to assess cow health during the study and not as a basis for comparison between treatments. Results are reported in supplementary material for all descriptive measures.

#### 2.5.1. Cow-Based Assessments 

Cow based assessments for hygiene (1 = clean, 5 = dirty) [27], locomotion (1 = sound, 5 = severely lame) [28], BCS (1 = very thin, 5 = excessively fat) [29] and weight were recorded at dry off, calving, 7 d postpartum and 14 d postpartum, by the same researcher. Hygiene and BCS were evaluated in the lactating pen before dry-off and in a palpation chute after calving. Locomotion was evaluated as the cow walked down a concrete exit alley from the parlor. Weight was measured using a scale (Tru-Test Limited, Mineral Wells, TX, USA) located in the exit alley from the parlor.

#### 2.5.2. Physiological Measurements 

A representative milk sample was collected throughout the milking by diversion of milk while it was harvested, into a collection bottle on d 0, 1 and 2 postpartum. Samples were analyzed for SCC, fat and protein at the University of Tennessee DHIA Lab (Knoxville, TN, USA). Daily milk production was collected using the automated milking system for 90 d.

To assess energy balance, blood was collected in serum collection tubes on d 0, 2, 5, 8, 11 and 14 postpartum, after milking between 900 h and 1000 h and immediately processed. Test strips (Precision Xtra β-ketone, Abbott Diabetes Care, Abingdon, UK) were dipped into the blood and analyzed for β-hydroxybuterate using the Precision Xtra meter (Abbott Diabetes Care, Abingdon, UK) previously validated [30].

#### 2.5.3. Cow Health 

Animal health assessments were performed during the first 7 d postpartum by the herd manager. Postpartum illnesses, including ketosis, clinical mastitis, metritis, retained placenta, displaced abomasum, prolapsed uterus and death were diagnosed subjectively by the herd manager and recorded as having, or not having had the illness during the 7 d period.

### 2.6. Statistical Analyses

The observational unit of this study was the cow and the experimental unit was the cow. Data was analyzed using the PROC MIXED procedure of SAS (SAS 9.3, SAS Int., Cary, NC, USA). Explanatory variables included: treatment (pasture or freestall), period (far-off, close-up, calving, or postpartum); and treatment × period to analyze lying and activity behaviours, including daily steps, daily lying time and daily lying bouts. Cows within treatment; and cows within treatment and period, were considered as random terms. Respiration rates were analyzed using treatment, THI and treatment × THI as explanatory variables. Feed bunk displacements during 2 h periods immediately following fresh feed delivery were analyzed using treatment, feed bunk stocking density and their interactions as explanatory variables. Intramammary infections were analyzed using treatment, sample day (d 0, 1, or 2 postpartum) and treatment × sample day to evaluate *Streptococcus agalactiae*, coagulase positive *staphylococci* and gram negative species. Results from statistical models are reported as least squares means (± SE). Hygiene score, locomotion score, BCS, body weight, BHBA concentration, postpartum milk production, SCC, milk protein, milk fat and colostrum quality; and quantities were averaged and reported as mean (± SD) for descriptive purposes.

## 3. Results

### 3.1. Descriptive Measures

Cow-based assessments, physiological measures and cow health are reported in the supplement.

### 3.2. Behavioural Assessment

Step data was not included for three pastured cows due to inaccurate data. A treatment × period interaction existed for daily lying time (*p* < 0.01; DF: 76; Figure 3) and daily lying bouts (*p* = 0.02; DF: 76; Figure 4), but not for the lying bout duration (*p* = 0.79; DF: 76; Figure 5) or daily steps (*p* = 0.18; DF: 67, Figure 6). Freestall housed cows lay down for longer during far-off and close-up periods (*p* < 0.01; DF: 76), but no differences occurred during calving (*p* = 0.60; DF: 76) and postpartum (*p* = 1.00; DF: 76) compared to pastured cows. Pastured cows lay down more frequently during calving (*p* < 0.01; DF: 76) compared to freestalls, though no differences occurred during far-off (*p* = 0.35; DF: 76), close-up (*p* = 0.39; DF: 76) or postpartum (*p* = 0.69; DF: 76). Period significantly affected the lying bout duration (*p* < 0.0001; DF: 76). Longer lying bouts occurred during far-off (90.7 ± 3.1 min/bout), followed by the close-up period (83.4 ± 3.2 min/bout), with lengths decreasing postpartum (73.2 ± 3.3 min/bout; *p* < 0.0001; DF: 76). Cows lay down for the shortest bouts during calving (47.1 ± 5.0 min/bout; *p* < 0.0001; DF: 76). Pastured cows took more steps during the study period than freestall housed cows (2714.9 ± 166.3 vs. 1835.0 ± 137.2 steps/d; *p* < 0.001; DF: 23). Cows took more steps on the day of calving (2888.7 ± 178.9 steps/d), followed by the far-off period (2416.2 ± 131.6 steps/d; *p* ≤ 0.01; DF: 67). Cows took the fewest steps during the close-up (1943.1 ± 134.2 steps/d) and postpartum periods (1851.7 ± 136.6 steps/d; *p* ≤ 0.01; DF: 67). Respirations rates increased with increasing THI when cows were managed on pasture compared to freestalls (*p* < 0.01; DF: 191; Figure 7). Increased feed bunk stocking density increased the number of feedbunk displacements more rapidly with freestall cows compared with pasture (*p* < 0.01; DF: 53; Figure 8).

### 3.3. Intramammary Infections

Freestall and pasture did not differ for growth of *Streptococcus agalactiae* (2051 ± 1392 vs. 22 ± 1358 cfu/mL, respectively; *p* = 0.31), coagulase positive *staphylococci* (282 ± 177 vs. 113 ± 174 cfu/mL, respectively; *p* = 0.50) and gram-negative species (808 ± 549 vs. 2 ± 536 cfu/mL, respectively; *p* = 0.30) in milk.

## 4. Discussion

The objectives of this study were to determine the effects of the dry cow management system (pasture or confined) on: (1) lying behaviour and activity; (2) feeding and heat stress behaviours; (3) intramammary infections, postpartum. In the present study, cows spent less time lying and took more steps during the dry period and lay down more frequently at calving on pasture, compared to freestalls. Pastured cows had greater respiration rate with increasing THI but fewer feedbunk displacements around feeding compared to freestall housed cows. Intramammary infections did not differ between the treatments.

### 4.1. Behavioural Assessment

Pastured cows were more active during the far-off period than the freestall cows. These results were similar to other studies where lactating cows spent 12.3 h per day lying in freestalls compared to 10.9 h per day for lactating cows lying on pasture [6], or 1.6 h per day more for lying in freestalls compared to pasture (daily lying time not reported) [9], likely due to the motivation to graze. This difference may be associated with the differences in time budget and priorities of cows on pasture compared to freestalls due to reduced clustering of resources [31]. When deprived of both resting and feeding, cows will choose to lie down, suggesting that cows seek to maintain a certain amount of lying down time within a day [32,33]. As resources are further to travel to, cows have a greater desire to graze and this may manipulate the desired time of resting pastured cows require when compared to freestall cows.

Freestall housed cows lay down more during the close-up period when compared to pastured cows, but the lying bouts did not differ and both groups reduced daily steps from the far-off period to the close-up period, though pastured cows had a greater number of daily steps throughout the study period. Resources, such as water and feed, were further away in a pasture system and required more travel in order to use those resources. Additionally, cows on pasture had the opportunity to graze. Cows grazed for 572 min when only offered a concentrate supplement to pasture compared to 252 min when offered a TMR supplement to pasture [5]. Grazing requires an increase in walking and thus, increases daily steps. However, there was a decrease in daily steps from the far-off to the close-up periods. In the current study, pastured far-off cows were fed a concentrate supplement while close-up pastured cows were fed a TMR supplement. Feeding additional concentrate to pastured mid-lactation cows determined that pasture equaled 60% of lactating cow’s total DMI [5]. When fed a TMR, pasture equaled 30% of lactating cow’s total DMI [5]. The reduction in daily steps when cows entered the close-up pen may be attributed to the additional TMR supplementation, which may have reduced the time they spent grazing.

No differences in lying time existed between freestall and pasture managed cows at calving. Lying bouts frequency increased at calving in both treatments however, cows lay down more frequently on the day of calving on pasture when compared to freestalls. Calving increased frequency of lying bouts at calving, compared to pre- and postpartum frequencies (17.3, 11.7 and 13.1 bouts/d, respectively) [34]. Calving discomfort is likely responsible for the increase in the lying bout frequency [35,36] and pasture may allow cows to more comfortably change lying position than when in a freestall calving pen.

Some advantages or disadvantages of increased activity during the dry period were previously investigated. Late gestation dry cows exercised using a treadmill had decreased heart rate and plasma lactate when regularly exercised compared to cows not exercised [34]. Reduced plasma lactate values indicate improved anaerobic metabolism and reduced metabolic challenge [34]. This may be particularly beneficial in times when muscles go into anaerobic metabolism, such as calving. In the current study, pastured cows were more active and providing access to pasture may be a natural alternative to implement exercise in the dry period and warrants further investigation.

Heat stress is characterized by an increase in rectal temperature and respiration rate [35]. In the current study, increased THI impacted pastured cows more dramatically, noted through an increased rate of respiration rate with increasing THI. Cows in freestalls had access to shade and fans, which offered improved heat abatement while cows on pasture only had access to trees located on the fence line, leading to greater susceptibility to become heat stressed and an increased behavioural response to heat. Respiration rate increases when THI rises above 73 [36]; however, mean THI was greater in freestalls when compared to pasture. Using fans and sprinklers during the dry period in the housing pen reduced respiration rate and rectal temperature and improved postpartum milk production [37,38], therefore the presence of fans may have prevented the increase of respiration rate in freestalls compared to pasture in the current study. Based on respiration rates, cows managed on pasture were more disposed to heat stress in the current study and housing cows in a freestall barn with heat abatement may reduce the effects of heat stress during the dry period.

Freestall housing increased the number of displacements with increasing feed bunk stocking density compared to pasture. Cows in freestalls had access to headlocks while those on pasture had access to a concrete bunk a wire barrier. Headlocks reduced the number of feed bunk displacements because the neck barrier allowed for protection [39], which is contrary to the results of the current study. However, since cows on pasture had access to grazing, which would constitute some of the cow’s diet, cows may have been less motivated to displace other cows for feed bunk space. It should also be noted that both treatments had a mean feed bunk stocking density below that of the recommended feed bunk stocking density, with feed bunk stocking density exceeding 100% on only five observational days on pasture and no days in freestalls during the study period.

Both freestall housing and pasture offer advantages and disadvantages during the dry period however, the system which is best suited depends on management. Though pasture increased heat stress behaviour during the dry period, additional heat abatement strategies implemented within the paddock may have helped reduce that behavioural response. Similarly, while freestall systems may increase the risk for feedbunk displacements, managing the herd to keep stocking density low during time of high feedbunk occupancy, may help elevate the issue. Either management system can work assuming that management is suited to handle the challenges associated with the particular system.

### 4.2. Intramammary Infections

Treatment did not affect intramammary infections. Pasture grazed dairy heifers had greater risk for postpartum subclinical mastitis when heifers had a prepartum infection, low teat height above the ground, or poor udder hygiene [40]. In the current study, teat height and prepartum mastitis were not recorded but hygiene between freestall and pasture treatments were similar and stocking densities were low throughout the study period, which may explain the similarity between intramammary infections postpartum. Previous studies note a reduced risk of mastitis when managed on pasture in heifers [41] and lactating dairy cows [42] but larger animal numbers were used and may be necessary to determine treatment differences.

### 4.3. Study Limitations

Limitations exist in the current study, including behavioural observations and group size. Two consistent observers monitored respiration rate and feeding behaviour during the length of the study. One observer trained the second in watching flank movements for respiration rate measurement and successful displacement criterion for feeding behavior. High inter-observer reliabilities have been previously reported for feeding behaviour [43,44,45] and respiration rate [24,25,46], indicating these measures can be reliably measured by multiple people with small variability. Though having the measure for this study would have been advantageous, the high level of previously reported reliability and training method likely ensured that observers did not create extreme variance in observations. 

Management type and stocking density may also represent confounding factors in this study. Pasture was the typical management practice of the research farm, meaning that additional animals were added to the pasture pens but not into the freestall pens. This had an impact on the feed bunk stocking density, which was much more variable in the pasture treatment. However, though feed bunk stocking density was more variable in pastured cows and often higher, this did not elicit more aggression at the bunk, which is likely related to feeding management.

## 5. Conclusions

Cows managed on pasture during the dry period lay down less and were more active when compared to the freestall housed cows. The lying bout frequency increased around calving, especially for cows managed on pasture, providing a management technique for dairy producers to better monitor the initiation of calving. Pasture may dispose cows to increased heat stress compared to freestall housed cows, indicated by increased respiration rates, demonstrating freestall housing may reduce exposure to heat stress during the dry period. Pasture also reduced the number of feed bunk displacements around feed delivery, potentially due to the availability of grass, reducing the motivation to displace cows from feed bunk space. Understanding the benefit of increased activity over lying time needs to be established for improved management of dry cows.

## Figures and Tables

**Figure 3:**
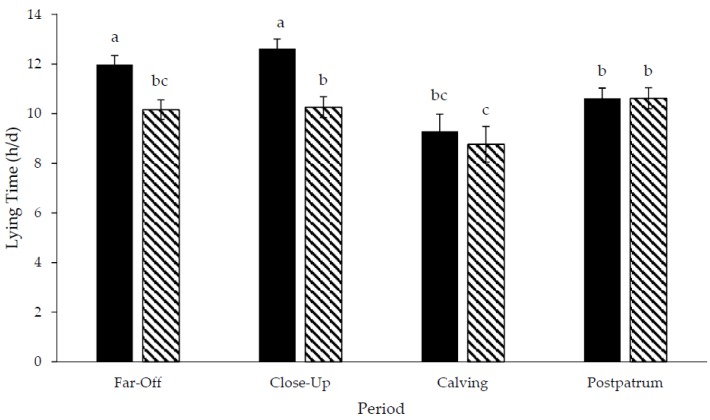
Lying time of Holstein cows managed in freestalls (solid bars; *n* = 14) or on pasture (striped bars; *n* = 14) during the dry period and early postpartum. Far-off: 60 to 15 d prepartum; close-up: 14 to 1 d prepartum; calving: calving date; postpartum: 1 to 14 d postpartum. ^a,b,c^ Different superscripts denote tendencies for differences between and within treatments (*p* < 0.10).

**Figure 4:**
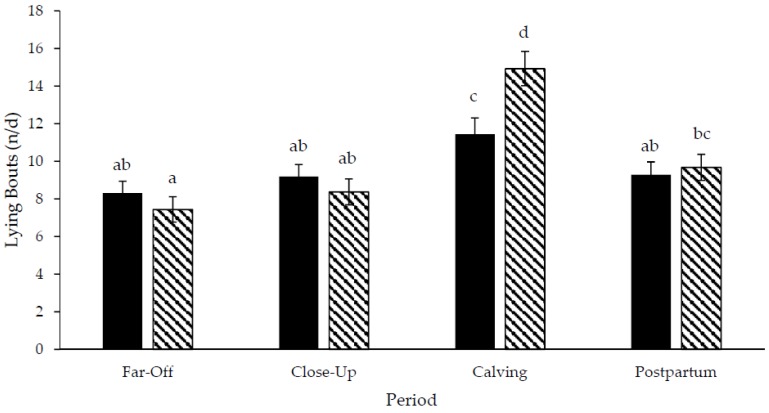
Lying bouts of Holstein cows managed in freestalls (solid bars; *n* = 14) or on pasture (striped bars; *n* = 14) during the dry period and early postpartum. Far-off: 60 to 15 d prepartum; close-up: 14 to 1 d prepartum; calving: calving date; postpartum: 1 to 14 d postpartum. ^a,b,c,d^ Different superscripts denote differences between and within treatments (*p* < 0.05).

**Figure 5:**
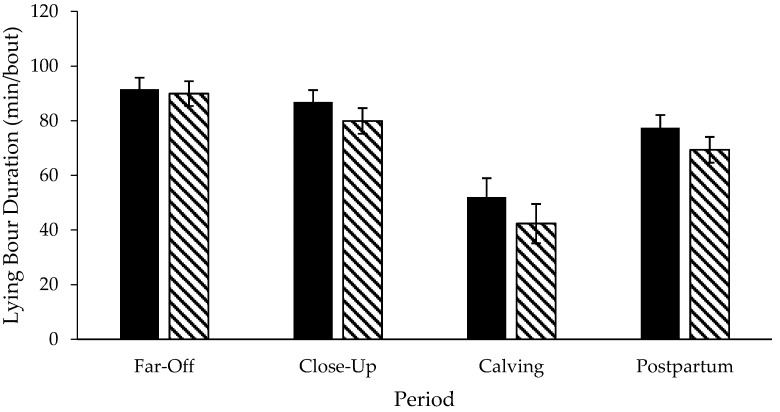
Lying bout duration of Holstein cows managed in freestalls (solid bars; *n* = 14) or on pasture (striped bars; *n* = 14) during the dry period and early postpartum. Far-off: 60 to 15 d prepartum; close-up: 14 to 1 d prepartum; calving: calving date; postpartum: 1 to 14 d postpartum.

**Figure 6:**
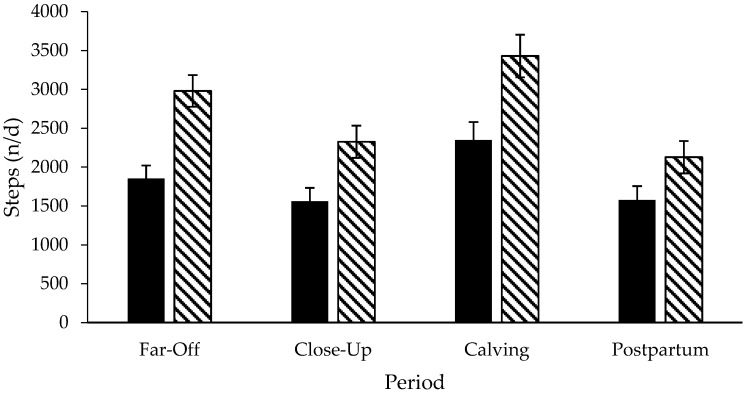
Daily steps of Holstein cows managed in freestalls (solid bars; *n* = 14) or on pasture (striped bars; *n* = 14) during the dry period and early postpartum. Far-off: 60 to 15 d prepartum; close-up: 14 to 1 d prepartum; calving: calving date; postpartum: 1 to 14 d postpartum.

**Figure 7:**
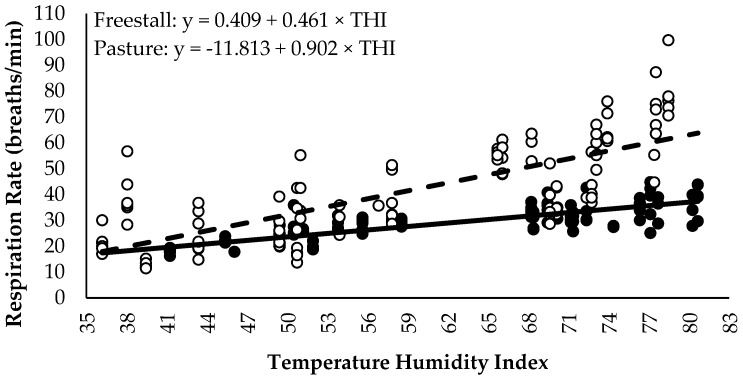
Predicted respiration rate by temperature-humidity index (THI) from respiration rates collected at 10 min intervals between 1300 and 1500 h for freestall (solid line and closed circles; *n* = 14) and pasture (dashed line and open circles; *n* = 14) treatments (*p* < 0.01).

**Figure 8:**
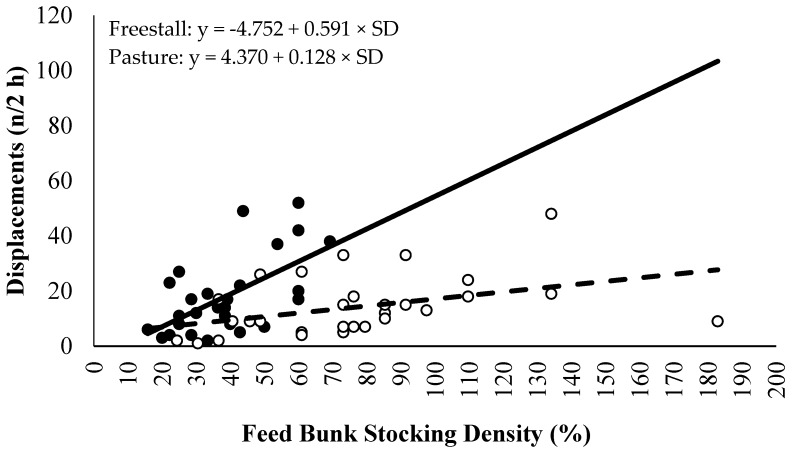
Model for number of feed bunk displacements during the 2 h period immediately after fresh feed delivery for freestall (solid line and closed circles; *n* = 14) and pasture (dashed line and open circles; *n* = 14) treatments (*p* = 0.01) by stocking density (SD).

**Table 1:** Nutrient analyses of pastures, hay, grain and corn silage fed during the study period on a dry-matter basis.

Feed	Pen ^1^	DM, %	CP, %	ADF, %	aNDF, %	Ca, %	P, %	K, %	Mg, %	Crude Fat, %	Lignin, %	Ash, %	TDN, %	NEL, Mcal/lb	NEM, Mcal/lb	NEG, Mcal/lb
Grass	A	25.1	14.2	44.1	76.3	0.58	0.26	1.34	0.44	2.17	5.01	10.5	64	0.66	0.62	0.33
Hay	A	90.6	11.2	44.8	73.2	0.45	0.26	2.28	0.34	1.76	4.48	9.90	62	0.64	0.59	0.31
Grass	B	26.0	15.1	40.9	72.0	0.60	0.26	1.57	0.41	2.40	3.98	9.51	67	0.69	0.67	0.38
Hay	B/C	91.2	11.7	43.2	72.0	0.51	0.26	2.10	0.34	2.00	4.65	7.95	63	0.65	0.61	0.33
Grass	C	24.6	14.8	40.5	71.5	0.60	0.26	1.53	0.40	2.44	3.80	9.40	68	0.70	0.68	0.39
Grain A		NR	23.0	15.7	NR	1.00	0.55	NR	NR	3.50	NR	NR	NR	NR	NR	NR
Grain B		NR	24.1	8.1	14.8	1.93	0.62	1.02	0.59	4.28	NR	NR	NR	0.77	NR	NR
Corn Silage		32.4	8.1	20.5	35.6	0.22	0.22	0.94	0.17	3.7	2.30	4.12	79	0.84	0.86	0.57
Pre-Mix Hay		89.5	9.1	36.9	59.6	0.33	0.22	1.71	0.17	2.1	2.2	8.48	61	0.55	0.56	0.30

NR = “Not Reported” by supplier (Grain A, Grain B).**^1^** Pen A contained 13 acres and housed far-off cows (60 to 15 d prepartum) from August to September 2013; Pen B contained 2.8 acres of pasture and housed far-off cows October 2013 to January 2014; Pen C contained 4.3 acres of pasture and housed close-up cows (14 d prepartum to calving) from October 2013 to January 2014; all pastures seeded with orchardgrass and KY-31 fescue mix.

**Table 2:** Temperature and humidity within freestall barn and on pasture.

Management System	Temperautre,°C	Range, °C	Relative Humidity, %	Range, %	THI	Range
Freestall	17.6 ± 8.6	3.5–30.8	56.3 ± 15.6	28.7–89.8	62.6 ± 12.2	41.2–80.7
Pasture	15.3 ± 9.6	−0.3–28.8	63.0 ± 15.7	35.0–93.8	59.0 ± 14.1	36.2–78.5

## Supplementary Materials

The following are available online at http://www.mdpi.com/2076-2615/6/7/41, Table S1: Cow-based assessment means ± SD of cows housed on either pasture (*n* = 14) or in freestalls (*n* = 14) during the dry period, Table S2: Physiological measure means ± SD of cows housed on either pasture (*n* = 14) or in freestalls (*n* = 14) during the dry period, Table S3: Postpartum health disorder incidences of cows housed on either pasture (*n* = 14) or in freestalls (*n* = 14) during the dry period.
