# Supplementary material for: A Case Study of Behaviour and Performance of Confined or Pastured Cows During the Dry Period
Source: Animals (Basel). 2016 Jul 13;6(7):41. doi: 10.3390/ani6070041 (PMC4961997; doi:10.3390/ani6070041)
Supplement: Supplementary file 1 [file animals-06-00041-s001.pdf]

Supplementary Materials: A Case Study of Behaviour and Performance of Confined or Pastured Cows during the Dry Period

Randi A. Black and Peter D. Krawczel

Table S1. Cow-based assessment means ± SD of cows housed on either pasture (n = 14) or in freestalls (n = 14) during the dry period.

Parameter	Day							
	DO		CA		PP1		PP2	
	Freestall	Pasture	Freestall	Pasture	Freestall	Pasture	Freestall	Pasture
Hygiene score [1]	2.07 ± 0.27	2.14 ± 0.36	3.00 ± 0.68	2.79 ± 0.89	2.85 ± 0.80	2.64 ± 0.93	2.42 ± 0.67	2.36 ± 0.50
Locomotion score [2]	1.36 ± 0.63	1.57 ± 0.65	1.64 ± 0.74	1.93 ± 0.73	1.77 ± 1.10	1.93 ± 0.73	1.58 ± 0.79	1.64 ± 0.84
Body condition score [3]	3.41 ± 0.25	3.29 ± 0.41	3.25 ± 0.23	3.06 ± 0.41	3.06 ± 0.25	2.95 ± 0.38	3.04 ± 0.30	2.80 ± 0.34
Weight, kg	668.6 ± 40.5	676.8 ± 95.0	695.8 ± 37.4	655.3 ± 79.0	651.3 ± 58.3	632.9 ± 72.3	636.7 ± 48.1	619.1 ± 78.4

[1] Hygiene measured using scoring system by (Reneau et al. [1]); [2] Locomotion measured using scoring system by (Flower and Weary [2]); [3] Body condition score measured using scoring system by (Wildman et al. [3]).

Table S2. Physiological measure means ± SD of cows housed on either pasture (n = 14) or in freestalls (n = 14) during the dry period.

Day	Housing							
	Freestall				Pasture			
	Milk Fat, %	Milk Protein, %	SCC, Cells/mL	BHBA, mg/dL	Milk Fat, %	Milk Protein, %	SCC, Cells/mL	BHBA, mg/dL
0	5.68 ± 2.60	8.79 ± 4.37	890,429 ± 1,022,770	0.48 ± 0.17	4.18 ± 1.34	8.63 ± 2.85	1,502,360 ± 1,458,420	0.46 ± 0.17
1	4.62 ± 1.95	6.08 ± 1.65	607,786 ± 670,639	-	3.88 ± 1.46	6.07 ± 1.43	1,035,640 ± 1,035,870	-
2	5.11 ± 1.58	5.42 ± 2.39	929,143 ± 1,789,920	0.62 ± 0.28	4.30 ± 1.14	5.28 ± 2.29	898,333 ± 1,234,870	0.55 ± 0.24
5	-	-	-	0.70 ± 0.29	-	-	-	0.64 ± 0.57
7	4.64 ± 1.08	3.83 ± 1.19	247,077 ± 526,620	-	4.13 ± 0.94	3.74 ± 1.09	222,893 ± 271,798	-
8	-	-	-	0.89 ± 0.70	-	-	-	0.43 ± 0.21
11	-	-	-	0.67 ± 0.69	-	-	-	0.39 ± 0.18
14	4.10 ± 0.60	3.11 ± 0.31	62,273 ± 123,725	0.58 ± 0.26	3.92 ± 1.45	3.17 ± 0.62	68,231 ± 75,654	0.41 ± 0.11

Table S3. Postpartum health disorder incidences of cows housed on either pasture (n = 14) or in freestalls (n = 14) during the dry period.

Housing	Health Disorder							
	Ketosis	Mastitis	Metritis	Retained Placenta	Displaced Abomasum	Prolapsed Uterus	Death	Total Cows [1]
Freestall, *n*	2	1	8	2	0	1	1	10
Pasture, *n*	1	1	5	3	1	0	0	6

[1] Total of number of cows which developed a health disorder during the study period.

References

1. Reneau, J.K.; Seykora, A.J.; Heins, B.J.; Endres, M.I.; Farnsworth, R.J.; Bey, R.F. Association between hygiene scores and somatic cell scores in dairy cattle. *J. Am. Vet. Med. Assoc.* **2005**, *227*, 1297–1301.
2. Flower, F.C.; Weary, D.M. Effect of hoof pathologies on subjective assessments of dairy cow gait. *J. Dairy Sci.* **2006**, *89*, 139–146.
3. Wildman, E.E.; Jones, G.M.; Wagner, P.E.; Boman, R.L.; Troutt, H.F., Jr.; Lesch, T.N. A dairy cow body condition scoring system and its relationship to selected production characteristics. *J. Dairy Sci.* **1982**, *65*, 495–501.

© 2016 by the authors; licensee MDPI, Basel, Switzerland. This article is an open access article distributed under the terms and conditions of the Creative Commons by Attribution (CC-BY) license (http://creativecommons.org/licenses/by/4.0/).
